# Anti-*Helicobacter pylori* and Urease Inhibition Activities of Some Traditional Medicinal Plants

**DOI:** 10.3390/molecules18022135

**Published:** 2013-02-07

**Authors:** Muhammad Amin, Farooq Anwar, Fauqia Naz, Tahir Mehmood, Nazamid Saari

**Affiliations:** 1 Department of Chemistry, University of Sargodha, Sargodha-40100, Pakistan; E-Mails: makhan1111@yahoo.com (M.A.); fauqia36@gmail.com (F.N.); tahiruosbiochem@yahoo.com (T.M.); 2 Faculty of Food Science and Technology, Universiti Putra Malaysia, 43400 UPM, Serdang, Selangor, Malaysia

**Keywords:** medicinal plants, anti-*Helicobacter pylori* activity, agar dilution method, urease inhibitory activity, Lineweaver-Burk plots, inhibition kinetics

## Abstract

Different parts of *Acacia nilotica* (L.) Delile, *Calotropis procera (*Aiton*)* W.T. Aiton, *Adhatoda vasica* Nees, *Fagoniaar abica* L. and *Casuarina equisetifolia* L. are traditionally used in folk medicine for the treatment of a variety of common ailments like nausea, cold, cough, asthma, fevers, diarrhea, sore throat, swelling, *etc*. The present study was aimed to evaluate the anti-*Helicobacter pylori* and urease inhibition activities of extracts produced from the above selected medicinal plants native to Soon Valley (home to an old civilization) in the Punjab province of Pakistan. Methanol, acetone and water extracts of the plants were evaluated for anti-bacterial activity against thirty four clinical isolates and two reference strains of *H. pylori*. Minimum inhibitory concentrations (MICs) of the extracts were determined using the agar dilution method and compared with some standard antibiotics like amoxicillin (AMX), clarithromycin (CLA), tetracycline (TET) and metronidazole (MNZ), used in the triple therapy for *H. pylori* eradication. *H. pylori* urease inhibition activity of the extracts was assessed by the phenol red method, wherein, Lineweaver-Burk plots were used to determine Michaelis-Menten constants for elucidating the mechanism of inhibition. Methanol and acetone extracts from *Acacia nilotica* and *Calotropis procera* exhibited stronger anti-*H. pylori* activity than MNZ, almost comparable activity with TET, but were found to be less potent than AMX and CLT. The rest of the extracts exhibited lower activity than the standard antibiotics used in this study. In the *H. pylori* urease inhibitory assay, methanol and acetone extracts of *Acacia nilotica* and *Calotropis procera* showed significant inhibition. Lineweaver-Burk plots indicated a competitive mechanism for extract of *Acacia nilotica*, whereas extract of *Calotropis procera* exhibited a mixed type of inhibition.

## 1. Introduction

It is now widely recognized that gastric and duodenal ulcers are generally caused by *H. pylori*, which survives and grows in acidic environments [1,2]. This organism releases urease that converts urea into ammonia and the released ammonia protects it from the acidic environment of the stomach. It has been demonstrated that a urease-negative mutant does not cause gastritis due to difficulties in colonization, therefore, specific inhibition of urease activity has been proposed as a successful strategy to eliminate the organism in the body [3]. The cases of *H. pylori*-related infections are increasing in the developing countries, while in some parts of the World more than 50% of the population is reported to be infected with *H. pylori* [1,2]. Triple therapy, comprising a proton pump inhibitor and any of the two antibiotics such as amoxicillin (AMX), clarithromycin (CLA), metronidazole (MNZ) and tetracycline (TET), is frequently employed to cure *H. pylori* infections [2]. Clinical trials in this aspect have demonstrated an eradication rate of about 80–90% by the use of a relevant triple therapy including AMX [4]. The success of commercially available drugs in the treatment of gastric ulcers is overshadowed by the various side effects associated with these drugs. There is also the resistance problem coming up by inappropriate and extensive use of such drugs [4], so there is a need to explore more effective anti-ulcer agents and urease inhibitors possessing enhanced efficacy against microorganisms while exhibiting less toxicity to human cells.

Plants have always been the main source of new drugs and folk medicines. Plants are well known to contain active metabolites, which are useful in treating various infectious diseases with no or less toxicity [5]. Several naturally occurring medicinal plants, herbs, and fruit extracts have been shown to possess antimicrobial activity against *H. pylori* [6]. Awareness is now growing regarding the preferred use of medicinal plant materials as prophylaxis and therapeutics over the synthetic drugs [7].

The Soon Valley, located in the Punjab province of Pakistan, is rich in medicinally important flora with significant potential for bioprospecting. Different parts of the plant *Acacia nilotica* (L.) Delile (*A. nilotica*), due to the presence of a wide array of secondary metabolites such as phenolics, alkaloids, terpenes, flavonoids and tannins [8], are known to exhibit anti-hyperglycemic, antimicrobial, molluscicidal, anti-hypertensive and anti-platelet aggregation, demulcent, styptic and astringent activities [9]. *Calotropis procera* (Aiton) W.T. Aiton, (*C. procera*) is a wild-growing plant having multifarious medicinal and biological properties [10]. Different parts of this plant are used in the treatment of leprosy, tumours, piles, diseases of spleen and abdomen [11]. *Fagonia arabica* L. (*F. arabica*) has traditionally been used for the treatment various diseases, namely hematological, neurological, endocrinological and inflammatory disorders, skin diseases, small pox and for endothermic reactions in the body [12]. *Adhatoda vasica* Nees, (*A. vasica*), an evergreen local medicinal plant, is commonly employed for the treatment of cold, cough, asthma and tuberculosis, *etc*. [13,14]. *Casuarina equisetifolia* L. (C. *equisetifolia*) is locally used as a folk remedy for the treatment of diarrhea, dysentery, stomach and nervous problems; anthelmintic and anti-diabetic properties have also been reported [15].

The previous literature revealed the isolation of urease inhibitors from some plants and herbs [16,17]. Typically, garlic extract is a natural inhibitor of urease [18]. *Hypericum oblongifolium* Wall has also been assayed for anti-urease activity [19]. Keeping in mind the need to identify more plant sources of urease inhibitors, we undertook the study to screen five regional plants abundantly available in the Soon Valley. To the best of our knowledge the subject plants have not been screened yet for their anti-*Helicobacter pylori* and urease inhibition activities. This provoked the need to carry out the present investigation. Different solvent extracts produced were screened for their anti-*H. pylori* activity by the use of agar dilution method. The extracts, which exhibited promising anti-*H. pylori* activity, were further tested for their urease inhibitory activity. The mechanism of inhibition was derived by drawing Lineweaver-Burk plots and the values of Michaelis-Menten constants (K_i_) were calculated from the slopes of each line in the plot.

## 2. Results and Discussion

### 2.1. Extraction Yield

Extraction yields of methanol, acetone and water extractable components from the selected medicinal herbs are given in Table 1. Amongst methanol extracts, M3 gave highest yield (35%), and M6 the lowest (13.5%). The order of yield for methanol extracts was found to be: M3 > M5 > M4 > M2 > M1 > M6 > M7. Of the acetone extracts, L3 gave the highest yield (36%) and L7 gave the lowest yield (14%). The order of yield for acetone extracts was: L3 > L5 > L4 > L2 > L6 > L1> L7. Similarly, amongst water extracts, K5 offered the highest yield (37%), while K6 the lowest (19%). The extraction yield order in this case was found to be K5> K3 > K4 > K7 > K2 > K1 > K6.

In the present investigation, the variation of the extract yields among different solvents and plant materials may be associated to the different chemical nature of the compounds present in these materials, as well as the polarity of the extraction solvents. The yields of extractable components, in addition to their chemical nature, are also strongly influenced by the concentration, polarity and nature of the extraction solvent, as well as the extraction technique employed. Therefore, an appropriate extraction system has to be employed to recover optimum contents of extractable antioxidant components. Typically, high polarity solvents such as methanol and ethanol are widely used to extract plants phenolic antioxidant components due to their compatibility and efficacy towards solubilization of such compounds [20].

### 2.2. *In Vitro* Anti-H. pylori Activity of the Extracts

The results for antibacterial activity (MICs values µg mL^−1^) of plant extracts, AMX, CLA, MNZ and TET, against 40 clinical and two reference strains of *H. pylori* are given in Table 2.

**Table 1:** List and extraction yields of indigenous medicinal plants, Soon Valley, Punjab, Pakistan.

Sr. No	Plant name	Family	Part used	Therapeutic application	Extract	% yield
1	*Acacia nilotica* (L.) Delile	Fabaceae	Leaves, flowers	Chewing of young leaves is quite effective against nausea. Flowers and Pods decoction is used as expectorant [8,9]	K_1_	21.0 ± 0.6
					K_2_	24.0 ± 1.2
					K_3_	33.0 ± 1.5
					K_4_	30.0 ± 1.2
2	*Calotropis procera* (Aiton) W.T. Aiton	Apocynaceae	Leaves, flowers	Powdered flowers are used in cold, cough and asthma. Leave juice is taken to relieve intermittent fevers [10,11]	K_5_	37.0 ± 1.2
					K_6_	19.0 ± 1.0
					K_7_	27.0 ± 1.0
					L_1_	17.0 ± 2.2
3	*Fagonia arabica* L.	Zygophyllaceae	Whole plant	decoction is effectively used against fever and also used as best blood purifier and as well as cooling agent [12]	L_2_	22.0 ± 1.0
					L_3_	36.0 ± 1.7
					L_4_	30.0 ± 1.5
					L_5_	31.0 ± 1.6
4	*Adhatoda vasica* Nees	Acanthaceae	Whole plant	Decoction of whole plant is used as remedy of all kinds of bronchial diseases [13,14]	L_6_	18.0 ± 1.8
					L_7_	14.0 ± 1.0
					M_1_	15.0 ± 0.9
5	*Casuarina equisetifolia* L.	Casuarinaceae	Fruit	A decoction from the astringent bark and fruit is used as a remedy for diarrhea, sore throat, cough and swellings [15]	M_2_	21.0 ± 2.0
					M_3_	35.0 ± 1.5
					M_4_	29.0 ± 1.6
					M_5_	32.0 ± 1.7
					M_6_	13.5 ± 1.8
					M_7_	14.3 ± 1.5

M: methanolic extract; L: acetone extract and K: aqueous extract series.

**Table 2:** Antibacterial activity (MICs values µg mL^−1^) of plant extracts, AMX, CLT, MNZ and TET, against 40 clinical and two reference strains of *H. pylori*.

Extracts	Range in clinical isolates	Range in reference strains	MIC_50_ in clinical isolates	MIC_50_ in reference strains	MIC_90_ in clinical isolates	MIC_90_ in reference strains
AMX	0.25–8	0.125–0.5	0.125–0.5	0.125	0.25	0.5
CLT	0.125–32	0.5–1	0.5	1	8	1
TET	1–64	4–8	8	4	32	16
MNT	1–512	16–32	32	32	32	32
L1	16–128	16–32	8	16	64	64
L2	8.00–128	32–64	64	128	128	128
L3	4.0–64	16–32	32	8	16	16
L4	32–256	32–64	128	256	512	256
L5	8.00–128	16–32	64	4	16	4
L6	128.0–1024	128–256	56	128	128	128
L7	64–512	16–32	32	16	32	16
M1	32–256	64–128	32	64	64	128
M2	8–128	32–64	32	16	32	32
M3	8–64	8–32	64	16	16	16
M4	32–256	16–32	128	16	32	32
M5	64–256	64–128	64	256	512	256
M6	128–512	128	256	256	512	512
M7	64–512	128	16	128	128	128
K3	8–64	16	64	8	32	32

The percent inhibition of *H. pylori* isolates at different concentration dose of the plant extracts and standard drugs is shown in Table 3.

**Table 3:** Efficacy of plant extracts against *H. pylori* clinical isolates.

Plant Isolate	Dose range µg mL^−1^ (% of *H. pylori* Isolates Inhibited)
L_1_	16–32 (36); 64–128 (64)
M_1_	32–64(40); 128–256(60)
L_2_	8.0–64 (64); 128 (36)
M_2_	8.0–64 (66); 128 (34)
L_3_	4–16 (72); 32–64 (28)
M_3_	8–16 (56); 32–64 (44)
L_4_	32–64 (52); 128–256 (48)
M_4_	32–64 (60); 128–256 (40)
L_5_	8–16 (52); 64–128 (28)
M_5_	64–128 (56); 256 (44)
L_6_	128–256 (20); 512–1024 (80)
M_6_	128 (16); 256–512 (84)
L_7_	64–-128 (28); 256–512 (72)
M_7_	64–128 (24); 256–512 (76)

Anti-*H. pylori* activity of AMX, CLA, TET and MNZ against the clinical isolates were in the range of 0.125–8.0 µg mL^−1^, 0.125–32.0 µg mL^−1^, 1.0–64.0 µg mL^−1^ and 1.0–512.0 µg mL^−1^, respectively, whereas against the reference strains they were in the range of 0.125–0.5 µg mL^−1^, 0.5–1.0 µg mL^−1^, 4.0–8.0 µg mL^−1^ and 16.0–32.0 µg mL^−1^, respectively (Table 2). The activities of L1 and M1 against local isolates were in the ranges of 16.0–128.0 µg mL^−1^ and 32.0–256.0 µg mL^−1^, respectively (Table 2) whereas the aqueous extracts exhibited activity within high range (>512, data not shown). Thus acetone extracts were found to be more potent than those of methanol. L1 and M1 exhibited MICs against reference strains in the ranges of 16.0–32.0 µg mL^−1^ and 64.0–128.0 µg mL^−1^, respectively. M2 and L2 exhibited almost similar activity (8.0–128.0 µg mL^−1^) against clinical isolates and reference strains. L3 was found to be more active against *H. pylori* local isolates with MIC in the range of 4.0–64.0 µg mL^−1^ while M3 exhibited slightly lower activity (MIC, 8.0–64.0 µg mL^−1^).

**Figure 1:**
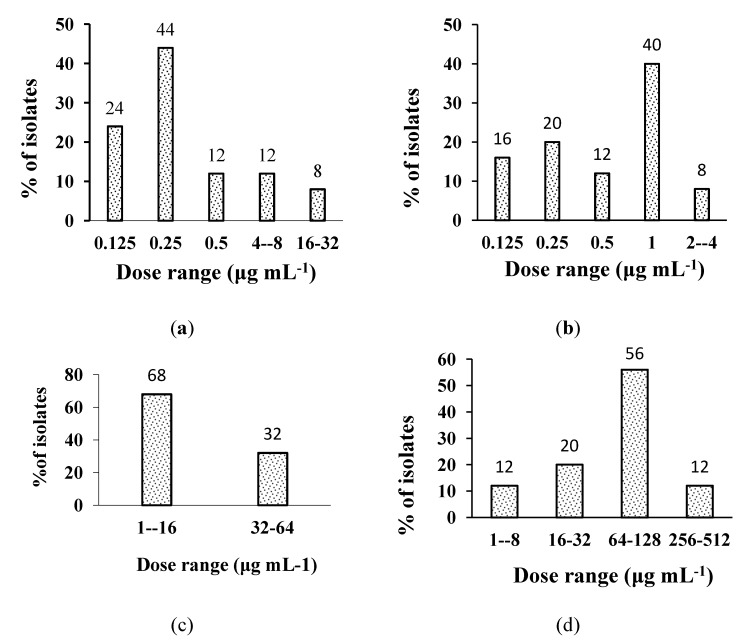
Effectiveness of (**a**) amoxicillin; (**b**) clarithromycin; (**c**) tetracycline; (**d**) metronidazole against clinical isolates of *H. pylori*.

Efficacy (% age inhibition of *H. pylori* local isolates at various activity ranges) of plant extracts is shown in Table 3, whereas for the standard drugs it is presented by Figure 1.

As far as percentage contribution of plant extracts against *H. pylori* clinical isolates is concerned, about 72% of the tested clinical isolates of *H. pylori* were found susceptible to L3 in the range 4.0–16.0 µg mL^−1^ and 28% in the range 32.0–64.0 µg mL^−1^. About 56% of the isolates were inhibited by M3 in the range of 8.0-16.0 µg mL^−1^ and 44% of the isolates were inhibited in the range of 32.0–64.0 µg mL^−1^ (Table 2). The activities of L2, L3, M2, M3 against reference strains were found to be in the range of 32.0–64.0 µg mL^−1^, 16.0–32.0 µg mL^−1^, 32.0–64.0 µg mL^−1^ and 8.0–32.0 µg mL^−1^, respectively. Both L4 and M4 exhibited activity against clinical isolates in the range of 32.0-256.0 µg mL^−^^1^, while L5 and M5 were active in the range of 8.0–128.0 µg mL^−1^and 64.0–256.0 µg mL^−1^, respectively (Table 2). About 52% of the isolates were inhibited by L5 in the range of 8.0-16.0 µg mL^−1^ followed by 28% in the range of 64.0-128.0 µg mL^−1^ whereas for M5 about 56% of the isolates were inhibited in the range of 64.0–128.0 µg mL^−1^ and 44% in the range of 256.0 µg mL^−1^. Therefore, L5 was proved more potent than M5.L6 and M6 were found to be more potent against tested clinical isolates in the range of 128.0–1,024.0 µg mL^−1^and 128.0–256.0 µg mL^−1^, respectively against the clinical isolates.

Referring to anti-*H. pylori* activity of the extracts it can be summed up that L3 and M3 exhibited stronger potential than MNZ, almost same activity as TET, however, were found less potent than AMX and CLA. On the other hand, M2 and L2 were found to be stronger anti-*H. pylori* agents than MNZ and less potent than other standard antibiotics used as positive controls in this investigation. The rest of the extracts also exhibited less activity than the standard antibiotics used in this study.

### 2.3. Urease Inhibitory Activity

**Figure 2:**
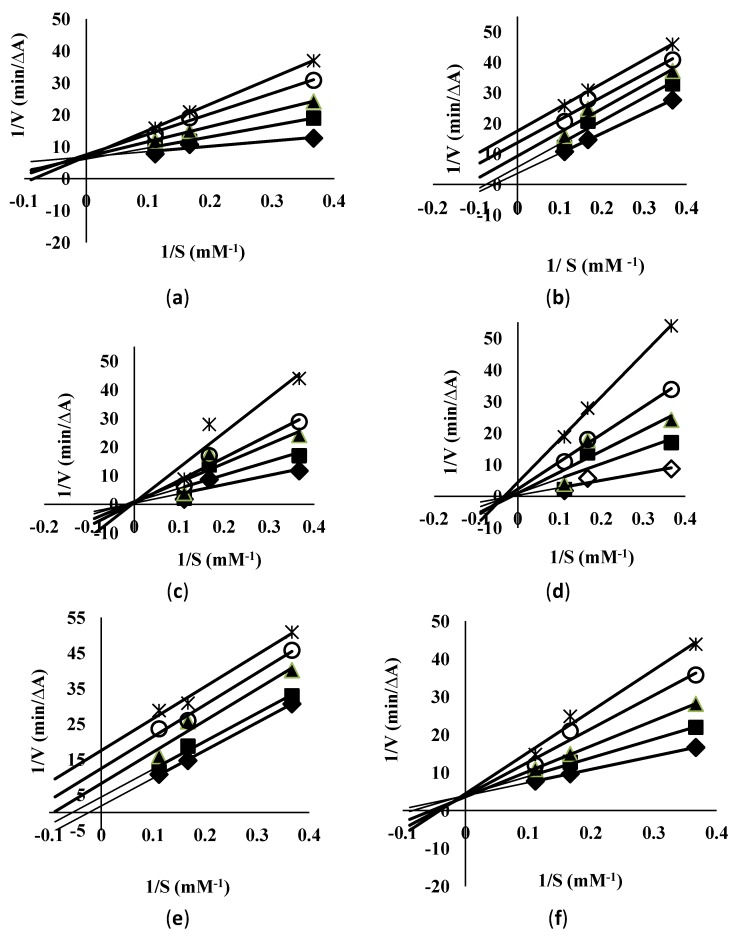
Inhibition of *H. pylori* urease by (**a**) L3; (**b**) L4; (**c**) L5; (**d**) M3; (**e**) M4; (**f**) M5; Lineweaver-Burk plots of the reciprocal of initial velocities vs reciprocal of four fixed substrate concentrations in absence (♦); presence of 80 mM (*); 60 mM (○); 40 mM (▲); 20 mM(■)of the substrate.

The results for the assessment of urease inhibitory activity (UIA) of the extracts are listed in Table 4. It was found that UIA increased linearly with the increase in the concentration of the extract. Especially, in the case of L_3_, M_3_, L_5_ and M_5_ Lineweaver-Burk plots and their replots indicated a non-competitive mechanism for L3 (Figure 2a), L5 (Figure 2c), M3 (Figure 2d) and M5 (Figure 2f) where the set of lines were found to intersect each other at the same point on x-axis depicting the same k_m_ values. The trend was found to be in accordance with those reported previously by Zarin *et al*. [21]. Therefore, it may be suggested that the mechanism was noncompetitive in which both the inhibitor and substrate were attached to the enzyme non-competitively.

On the other hand, L_4_ (Figure 2b) and M_4_ (Figure 2e) exhibited a mixed type of inhibition as a change in both V_max_ and affinity (K_m_ value) of urease toward the substrate (urea) was observed. Therefore, it could be predicted that in this case the inhibitor affected the affinity of the enzyme for its substrate, yet it did not bind at the active sites for that substrate.

**Table 4:** % Urease inhibitory activity (MIC) of plant extracts showing potent anti-*H. pylori* potential.

Sample Extracts	Concentration (µg mL^−1^)						
	4	8	16	32	64	128	256
L3	9.20 ± 0.29	19.45 ± 0.55	38.31 ± 0.45	72.22 ± 0.13	86.56 ± 0.12	-----	-----
L4	----	-----	-----	7.23 ± 0.22	19.21 ± 0.34	32.21 ± 0.46	58.21 ± 0.42
L5		9.33 ± 0.88	18.41 ± 0.66	36.21 ± 0.56	68.21 ± 0.15		
M3	8.21 ± 0.76	16.11 ± 0.89	33.23 ± 0.59	67.32 ± 0.75	88.21 ± 0.12	-----	------
M4	------	------	------	12.23 ±0.96	23.21 ± 0.46	39.12 ± 0.53	48.22 ± 0.24
M5	------	12.21 ± 0.23	24.51 ± 0.82	48.23 ± 0.52	86.21 ± 0.46	------	------

The values are mean ± SD of triplicate measurements.

### 2.4. Discussion

*H. pylori* is recognized as class 1 carcinogen by the WHO, consequently efforts are being focused worldwide for its eradication through the application of several therapies. In the present experiments, the plant extracts exhibiting excellent anti-*H. pylori* activity were further screened for their urease inhibitory activity. It is well accepted that *H. pylori* releases urease, a nickel-containing enzyme that transforms urea into ammonia (NH_3_) which protects *H. pylori* from the acidic environment of the stomach. The activity of urease can be controlled by the application of urease inhibitors as a viable strategy to inhibit *H. pylori* infections [22]. Urease inhibitors can play an important role in the eradication of infection caused by urease-producing bacteria. In this context, selected inorganic salts, heavy metal ions and synthetic organic compounds and antibiotics have been used as specific urease inhibitors [3,23]. Treatment of *H. pylori* using such synthetic compounds is associated with several problems such as high pretreatment cost, pretreatment bacterial resistance and adverse side effects. Therefore, exploration of some safer urease inhibitors derived from medicinal plants is becoming important as an alternate therapy against *H. pylori* based infections.

After initial screening at 1,024 µg mL^−1^, further MIC determination revealed that all of the extracts exhibited anti-*H. pylori* activity, except aqueous extracts. Furthermore, acetone extracts were found to be more active than methanol extracts. This may be due to the presence of higher concentration of active phytochemical constituents in the acetone extracts than that in aqueous and methanolic extracts. Amongst the plants tested, the extracts from *A. nilotica* and *C. procera* were found to be superior anti-*H. pylori* and urease inhibitory agents as compared to those from *A. vasica*, *F. arabica* and *C. equisetifolia.* The variation in anti-*H. pylori* activity of the extracts from *A. nilotica* leaves and flowers can be linked to the compositional difference of constituent phytochemicals between the two parts of this plant [24]. Anti-*H. pylori* activity of the L3, L5, M2 and M3 extracts were noted to be better than that of the standard antibiotic MNZ, almost same as TET, however, less than AMX and CLA. Therefore, these extracts appeared to be better anti-*H. pylori* agents than MNZ and TET due to their superior activity, lesser toxicity and cost effectiveness. The extracts can be suitable for treating the local isolates. The use of reference strains verify that local isolates are the strains related to the main class of the bacteria (*H. pylori*).

Pretreatment antibiotic resistance of *H. pylori* to MNZ and TET is alarmingly increasing in the developing countries [25]. This has been attributed to over the counter availability of these drugs in developing countries [4]. The bactericidal activity against different strains of Gram +ve and Gram –ve bacteria of various fractions isolated from the leaves of *A. nilotica* has already been reported [26]. Anti-*H. pylori* activity of *A*. *nilotica* leaves may be attributed to the hydrolysable tannins, saponins, glycosides, phenols, terpenes and flavonoids isolated from its leaves [27].

As far as the mechanism of urease inhibition is concerned, it was found to be noncompetitive in which inhibitor and substrate both were attached to the enzyme non-competitively. Antimicrobial activity of water extract from the bark of *A. nilotica* against some strains of bacteria and fungi reports has previously been investigated [28], but so far nothing has been reported on its anti-*H. pylori* activity. Our results clearly revealed a strong anti-*H. pylori* and urease inhibitory effect of A. *nilotica* which provoked further to perform a comprehensive isolation and characterization of active constitutes from this species as potent anti-*H. pylori* and urease inhibitory agents. The research on the isolation of active constituents of *A. nilotica* is in progress by our research group as a part of our systematic studies. Different parts of *C. procera* have widely been used in the Indo-Pakistani sub-continent for the treatment of leprosy, ulcers, tumors, piles and for the diseases of liver and abdomen [29]. The powder of the flowers of *C.procera* is used for the treatment of bleeding peptic ulcers [30], and the secretions from the root bark is traditionally used for the treatment of skin diseases and enlargements of abdominal viscera and intestinal worms [31]. Antibacterial activity of *C. procera* against Gram +ve and Gram −ve bacteria has already been demonstrated [10,11].

*F. arabica*, commonly known as akk in Soon Valley, is used for the treatment of fever [32] and some kinds of gastro-intestinal reflux diseases. In the present investigation, methanol extract of *F*. *arabica* (L1) was found to be more potent (MIC 16.0–128.0 µg mL^−1^) against *H. pylori* in contrast with M1 (acetone extract) (MIC 32.0–128.0 µg mL^−1^). However, both of the extracts exhibited stronger activity than MNZ while less than other standard antibiotics used in this study (Table 2). *F. arabica* extracts have already been reported for possessing antimicrobial properties against different strains of Gram +ve and Gram –ve bacteria [33]. Owing to its traditional uses in curing wounds, diaherrea and some symptoms of bleeding ulcers [32,33], this is of course for the first time that we have determined its antimicrobial activity against *H. pylori*.

The present study revealed that the tested plant extracts were able to display enzyme inhibition like a single compound and they may be utilized in curing various diseases and development of natural antibiotics. The sample extracts under investigation were found to exhibit good inhibitory properties especially in case of flowers of *A. nilotica* and *C*. *procera*, however, leaf extracts of *C. procera* showed mixed type inhibition. Generally, acetone extracts were noted to be more effective inhibitors as compared to methanol extracts.

Although *H. pylori* eradication by ingestion of medicinal plants has been controversial [34], it is plausible that active ingredients therein may suppress the pathogenicity of *H. pyl*ori by inhibiting its urease by various mechanisms. The findings of the present investigation may partially validate the use of these local herbs in gastric diseases but further bioactivity-guided scientific investigations are required to formulate suitable natural pharmaceuticals and antibiotics to combat with the *H. pylori* infections on clinical grounds.

## 3. Experimental

### 3.1. Collection of Plant Materials

The plants including *A. nilotica*, *C.procera*, *A. vasica*, *F.arabica* and, *C. equisetifolia* were collected from the southern rural areas (around Khora, Noshehra, Khabeki, Sodhi and Kanhati garden) of Soon Valley, Punjab, Pakistan, during the period 2010–2011. At least three different samples for each plant material were harvested. Further identification and authentication of the specimens was made by an expert taxonomist, Dr. Amin Shah at the Department of Biological Sciences, University of Sargodha, Pakistan. The detail of plants and their specific parts used in this work are listed in Table 1. The plant materials were dried under shade for 7 days and ground into fine powder. After sieving (80 mesh) they were transferred to air-tight polyethylene zipper bags, labeled and stored till further use. Voucher plant specimens were deposited in the Herbarium of the Department of Biological Sciences, University of Sargodha, Sargodha, under specimen number UOS-Chem-1148-11, for future reference. The solvents and other chemicals were of analytical grade (Fisher Scientific, Loughborough, UK).

### 3.2. Preparation of Plant Extracts

The powdered plant parts (5 g) were separately soaked in deionized water, methanol and acetone (50 mL) in a clean and dry reagent bottle covered with a lid at 37 °C for overnight. The samples were then shaken by use of an orbital shaking incubator (Model PA-42/250 R) at 45 °C and 150 rpm for four hours. The extracts thus obtained were filtered and the filtrates were centrifuged at 300 × *g* for about 20 min and evaporated under reduced pressure at 45 °C by the use of a Rotavapor^®^ R-210/R-215 rotary evaporator (Büchi Labortechnik AG. Flawil, Switzerland). The extraction yield was calculated as: % Yield = (weight of extract obtained/weight of powdered sample taken) × 100.

The aqueous extracts of *F. arabica* (whole plant), *A. nilotica* (leaves), *A. nilotica* (flowers), *C. procera* (leaves), *C. procera* (flowers), *C. equisetifolia* (fruits), and *A. vasica* (whole plant) were labeled as K_1_, K_2_, K_3_, K_4_, K_5_, K_6_, K_7_, respectively; methanol extracts as M_1_, M_2_, M_3_, M_4_, M_5_, M_6_ and M_7_ while acetone extracts as L_1_, L_2_, L_3_, L_4_, L_5_, L_6_ and L_7_ as mentioned in Table 1. The extracts were stored at −4 °C till further uses.

### 3.3. H. pylori Isolates

A total of 34 clinical isolates of *H. pylori* were obtained and characterized as reported earlier [3,19]. Two reference strains, NCTC 11637 and NCTC 11638, were procured from National Health Protection Agency, London, UK.

### 3.4. MIC Determination

Agar dilution method according to the guidelines provided by CLSI [35] was used for the determination of antimicrobial activity of the selected plant extracts against the standard strains and clinical isolates of *H. pylori*. The frozen clinical isolates were thawed and diluted using Muller Hinton infusion broth and adjusted to 0.5 Mc Farland standard (10^7^ cfu mL^−1^). A standardized loop full culture was used to seed the bacterial suspension onto the plates. For initial screening, plant extracts having concentrations 1,024 μg mL^−1^ were tested against 40 clinical and two reference strains of *H. pylori* by agar dilution method. Extracts showing 100% inhibition were subjected to determination of minimum inhibitory MICs. The wells on the plate were filled with two-fold serially diluted test extract and the standard drugs (AMX, CLA, MET and TET) having final concentrations of 1,024 to 0.125 μg mL^−1^. The control well was filled with dimethyl sulfoxide as a negative control. The dilutions were transferred to Mueller Hinton infusion agar supplemented with foetal bovine serum, campylobacter selective supplement Skirrow, SR 69 consisting of vancomycin (5 mg), polymyxin (1250 IU) and trimethoprim (2.5 mg), and inoculated with the test culture. The plates were incubated under microaerophillic set up at 37 °C for 72 or 96 h and MICs were then determined as per standard procedure. The break points to define a resistant strain were: metronidazole, ≥8.000 μg mL^−1^; clarithromycin, ≥1.000 μg mL^−1^; amoxycillin, ≥0.500 μg mL^−1^ and tetracycline, ≥16 μg mL^−1^ [2,36]. Triplicate experiments were performed.

### 3.5. Urease Inhibition Assay and Kinetics

#### 3.5.1. Isolation of *H. pylori* Urease

MHIB, supplemented with 10% fetal bovine serum was used as medium for the growth of *H.pylori* (NCTC-11638, Health Protection Agency, London, UK). The culturing was continued 24 h at 37 °C under microaerobic conditions (Campygen, Oxford, UK). The procedure described by Mao *et al*. [37] was used for the isolation of *H. pylori* urease. Briefly, the broth cultures (50 mL, 2.0 × 10^8^ CFU mL^−1^) were subjected to centrifugation (5,000 × *g*, 4 °C)and the recovered bacterial mass was washed twice using phosphate-buffered saline (pH 7.4) and then stored at −80 °C. Subsequently, *H. pylori* was thawed to ambient (room) temperature, followed by mixing with 3 mL of distilled water and protease inhibitors and sonication for 60 s. After centrifugation (15,000 × *g*, 4 °C), the supernatant was desalted by eluting through SephadexG-25 column (Pharmacia Bio-tech, Uppsala, Sweden). The resultant crude urease solution was mixed with an equal volume of glycerol and then preserved under refrigerator (4 °C) for further uses.

#### 3.5.2. Urease Inhibitory Assay

The reaction mixture comprising 55-µL phosphate buffer solution (3 mM, 4.5 pH), 25 µL of urease enzyme solution and test compound (5 µL, only those extracts which exhibited activity against *H. pylori*) were subjected to incubation for 15 min (30 °C) in 96-well plates. Urease activity was determined by measuring the ammonia released during the reaction [38]. In brief, 40 µL of each phenol reagent, containing a mixture of 1% phenol, 0.005% of sodium nitroprussside, and appropriate amount of alkaline (NaOH) reagents were added to each of the well. The absorbance of the final reaction mixture, after 50 min, was recorded at 630 nm, with a micro-plate reader. The results were computed using a built-in software of the microplate reader machine. Inhibition (%) were calculated after measuring optical density (OD) using the formula: 100 – (OD) test well/(OD) control. The control used was thiourea, while the IC_50_ values (the concentrations that inhibited the hydrolysis of the 50% substrate), were determined using EZ-Fit kinetic database (Perrella Scientific Inc., Amherst, NH, USA). The Lineweaver-Burk plots were recorded and the values for Michel-menton constants (K_i_) were determined using the slopes of each line plot. The kinetics was studied by taking reciprocal of enzyme activity along y-axis and reciprocal of substrate concentration along x-axis. The trend of lines for different concentrations of inhibitors gave the idea about the mechanism of inhibition. Three different concentrations of substrate (urea; 2.5 mM, 5.0 mM and 10.0 mM) were used for each extract sample.

## 4. Conclusions

The increasing development of antibiotic resistance in *H. pylori* against synthetic drugs is a worldwide concern. The use of medicinal plants and/or their chemical components may have potential benefits as anti-*H. pylori* agents for addressing such problems. The findings of the present investigation provide a scientific support towards the traditional uses of plant materials under study in stomach related diseases. Besides, an appreciable anti-urease activity exhibited by the tested plant extracts further supports their uses to inhibit *H. pylori* related infections. It is, therefore, concluded that these medicinal plants can be explored as viable sources to isolate some natural anti-*H. pylori* and urease inhibitory agents.
